# Predictive value of insulin resistance related indicators for osteoporosis risk in patients with type 2 diabetes mellitus: a real-world study

**DOI:** 10.3389/fmolb.2026.1795616

**Published:** 2026-07-22

**Authors:** Qikai Huang, Zhen Jian

**Affiliations:** Department of Orthopedics, Shanghai Pudong Hospital, Fudan University Pudong Medical Center, Shanghai, China

**Keywords:** insulin resistance, osteoporosis, quantitative CT, risk factor, tyg index, tyg-BMI, tyg-WC, type 2 diabetes mellitus

## Abstract

**Background:**

Insulin resistance (IR) contributes to osteoporosis (OP) by impairing bone microarchitecture, posing significant fragility-fracture risk in patients with type 2 diabetes mellitus (T2DM). The triglyceride–glucose (TyG) index and its anthropometry-adjusted derivatives (TyG–body mass index [BMI], TyG–waist circumference [WC]) are simple IR surrogates that may aid OP risk stratification. This study evaluated TyG, TyG-BMI and TyG-WC as independent predictors for OP in T2DM.

**Methods:**

In a retrospective cohort of 568 patients with T2DM (36 OP cases), baseline clinical, laboratory (HbA1c, haemoglobin [HGB], high-density lipoprotein cholesterol [HDL], serum calcium [Ca]) and bone-metabolism (procollagen type 1 N-terminal propeptide [P1NP], osteocalcin [OC], bone-specific alkaline phosphatase [BAP]) data were analyzed. Candidate covariates were selected by least absolute shrinkage and selection operator (LASSO) regression with variance inflation factors (VIF) screening. Because the events-per-variable ratio was low (≈4), three Firth’s penalised-likelihood logistic regression models were fitted for TyG, TyG-BMI or TyG-WC as the primary analysis. Discrimination was assessed by the area under the receiver-operating-characteristic curve (AUC) with 95% confidence intervals (CIs) and pairwise DeLong tests.

**Results:**

All three indices were significantly higher in OP than non-OP patients (all P < 0.001). After Firth-penalised adjustment, only remained independently associated with TyG-WC (OR 1.004; 95% CI 1.001–1.008; P = 0.013); TyG (P = 0.075) and TyG-BMI (P = 0.105) showed positive but non-significant associations. Higher HbA1c, OC and BAP increased the odds of OP, while male sex and higher HGB were protective. Discrimination was high and similar across models (apparent AUCs: 0.896 [TyG], 0.887 [TyG-BMI] and 0.901 [TyG-WC], with no DeLong-significant difference between indices.

**Conclusion:**

Among the three IR surrogates, only TyG-WC was independently associations with OP in T2DM, although overall discrimination was comparable across indices. TyG-WC may serve as low-cost adjuncts for identifying diabetic patients at elevated OP risk, pending prospective external validation.

## 1 Introduction

Osteoporosis (OP) is a systemic metabolic bone disorder characterized by compromised bone microarchitectural, posing a substantial fragility-fracture burden on the aging population (Ravindran et al., 2025; Qaseem et al., 2017; Kushchayeva et al., 2022; Curry et al., 2018). This burden is disproportionately amplified in patients with type 2 diabetes mellitus (T2DM); epidemiological estimates indicate that 28% of diabetic patients exhibit coexisting OP (Liu et al., 2023). Importantly, diabetic skeletal fragility is a sex-sensitive condition: while postmenopausal females inherently face elevated OP risk, T2DM disproportionately increases fracture risk in both sexes, often independent of preserved bone mineral density (BMD), underscoring the need for sex-aware metabolic risk stratification (Cooper et al., 2021; Papaioanno et al., 2021; Pan et al., 2023).

Insulin resistance (IR) is key to the pathophysiology of T2DM and is increasingly recognized as a mechanistic link between dysglycemia and skeletal impairment (Yaribeygi et al., 2019; Kuo and Chen, 2017). While the hyperinsulinemic-euglycemic clamp remains the gold standard for assessing insulin sensitivity, its invasive nature, high cost, and time-consuming protocol render it unfeasible for large-scale epidemiological screening and routine clinical assessment, necessitating reliable, accessible surrogate markers (Karelis et al., 2004). The triglyceride–glucose (TyG) index, defined from fasting triglyceride and glucose concentrations, has been validated as a convenient marker of IR (Kurniawan, 2024; Ramdas et al., 2022). And has been associated with multiple IR-related conditions including cardiovascular disease, cerebrovascular disease, hepatic steatosis, renal dysfunction and neuropsychiatric disorders (Zhou et al., 2023; Saffar et al., 2025; Huo et al., 2024; Chen et al., 2024; Zhang et al., 2025; Liu et al., 2024). Emerging evidence further links TyG to bone outcomes: higher TyG has been associated with fragility fractures in patients with diabetes and with alterations in bone-turnover markers among patients with osteoporotic fracture (Pan et al., 2023; Li et al., 2024; Xu J. et al., 2024).

Mechanistically, IR, adiposity and bone metabolism form a pathophysiological triad in T2DM (Kahn et al., 2006; Gkastaris et al., 2020). IR directly impairs osteoblast differentiation and promotes chronic low-grade inflammation (Ravindran et al., 2025). Concurrently, IR-driven visceral adiposity—a hallmark of T2DM—acts as an active endocrine organ, secreting proinflammatory cytokines (e.g., TNF-α, IL-6) and dysregulated adipokines (e.g., decreased adiponectin) that disrupt the bone microenvironment, stimulating osteoclastogenesis and suppressing bone formation (King et al., 2022). Thus, adiposity and IR synergistically drive diabetic bone fragility, suggesting that indices capturing both metabolic defects may better reflect skeletal risk than IR measures alone.

To capture this combined burden, composite indices such as TyG-BMI and TyG-WC, have been developed. These modified indices have demonstrated improved performance over TyG alone for predicting IR and several metabolic disorders, including hypertension and nonalcoholic fatty liver disease, in multiple cohorts (Tian et al., 2024; Wen et al., 2022; Er et al., 2016; Song et al., 2025).

However, it remains mechanistically and clinically unclear whether these adiposity-adjusted IR indices better capture the synergistic detrimental effects of IR and visceral fat on bone microarchitecture in T2DM than TyG alone. Existing studies on TyG-related indices and bone health in T2DM are inconsistent and lack comparative analyses to identify the optimal predictive model (Li and Zan, 2024; Xu MJ. et al., 2024).

Based on the above, the present study aimed to systematically evaluate and compare the independent predictive values of TyG, TyG-BMI and TyG-WC for OP in a clinical T2DM cohort. We hypothesized that indices integrating central adiposity would more accurately capture the IR-adiposity-interlinked bone fragility, thereby providing a simple and effective tool for early OP risk stratification in diabetic patients.

## 2 Methods

### 2.1 Study design

This study was a single-center, retrospective observational study, with a cross-sectional analytical design based on baseline data extracted from the electronic medical record (EMR).

### 2.2 Study population

Consecutive patients with T2DM admitted to the endocrinology department of hospital from 1 June 2023 to 30 June 2025 were enrolled. The study population was prespecified as T2DM only, for three biological and design reasons. First, T2DM is the clinical phenotype most strongly hypothesized by chronic IR, whereas type 1 diabetes mellitus (T1DM) is driven by absolute insulin deficiency, in which the TyG index reflects an entirely different physiological state and is not a valid IR surrogate. Second, T1DM bone disease primarily manifests as low bone formation due to deficient insulin like growth factor-1 (IGF-1) signalling from insulin loss, and is biologically distinct from the IR-adiposity-mediated phenotype examined here. Third, gestational and other secondary forms of diabetes involve transient or pharmacologically induced glucose dysregulation in heterogeneous hormonal contexts, are uncommon in our adult endocrinology ward, and would introduce substantial confounding.

#### 2.2.1 Inclusion criteria

(1) Age ≥18 years; (2) physician-confirmed T2DM by the Chinese Diabetes Society (2025). (3) complete demographic, anthropometric, biochemical and lumbar quantitative computed tomography (QCT) bone mineral density (BMD) data.

#### 2.2.2 Exclusion criteria

(1) Known secondary OP (primary hyperparathyroidism, Cushing’s syndrome, multiple myeloma, hyperthyroidism); (2) chronic diseases or drug exposure substantially affecting bone metabolism, including severe hepatic or renal insufficiency, inflammatory bowel disease, rheumatoid arthritis, long-term corticosteroid use (prednisone equivalent ≥5 mg/day for >3 months), anti-epileptic drugs, or supratherapeutic thyroid hormone; (3) prior fragility fracture or current treatment with anti-OP drugs (bisphosphonates, denosumab, teriparatide, romosozumab.); and (4) missing essential data or biologically implausible values. Missing data were defined as absence of any one of: fasting triglyceride (TG), fasting plasma glucose (FPG), BMI, WC, age, sex, or lumbar QCT-BMD. Biologically implausible values were defined *a priori* as BMI <10 or >70 kg/m^2^, age <18 or >120 years, or laboratory values outside three orders of magnitude of the published reference range, indicating likely data-entry error.

### 2.3 Sample size

As a single-centre retrospective study, all consecutive eligible patients during the study period were included (all-comer design); no *a priori* sample-size calculation was performed. The final analytic sample comprised 568 patients with 36 OP cases. With nine covariates in each multivariable model, the events-per-variable (EPV) ratio was approximately four, below the conventional threshold of ten recommended for maximum-likelihood logistic regression (Peduzzi et al., 1996). To minimise the small-sample bias and the risk of separation that arise at low EPV, Firth’s penalised-likelihood logistic regression (Heinze and Schemper, 2002) was prespecified as the primary analysis (Section 2.8.3). A *post hoc* statistical-power estimate for the achieved sample, based on the method of Hsieh et al. (Hsieh et al., 1998), is reported in the Supplementary Methods (Liu et al., 2023).

### 2.4 OP diagnosis

Lumbar (L1–L3) volumetric BMD was measured by quantitative computed tomography (QCT) using the Mindways QCT Image Analysis system (Mindways Software, Austin, TX, USA). OP was defined, according to the Chinese guideline for the diagnosis of OP with QCT (2018) (The committee for the Chinese guideline for the diagnosis of osteoporosis with quantitative computed tomography, 2019), as lumbar volumetric BMD ≤80 mg/cm^3^ averaged across L1–L3; values >80 mg/cm^3^ defined the non-OP group.

### 2.5 Anthropometric and clinical measurements

Body weight, height, waist circumference (WC, measured at the midpoint between the lowest rib and iliac crest at end-expiration), systolic and diastolic blood pressure, and pulse rate were recorded by trained nursing staff on admission. BMI was computed as weight (kg)/height (m) (Qaseem et al., 2017).

### 2.6 Biochemical and haematological markers

Venous blood (5 mL total) was drawn from a peripheral vein on the morning after admission, after a minimum 8-h overnight fast. Samples were collected into evacuated tubes (3 mL serum-separator tube for biochemistry, 1 mL EDTA tube for HbA1c (glycosylated hemoglobin), 1 mL EDTA tube for complete blood count) and transported to the central laboratory within 1 h. Serum was separated by centrifugation at 3,000 *g* for 10 min at 4 °C and analysed within 2 h of collection on an automated platform (Roche cobas 8000 c^70^2 modular analyser; Roche Diagnostics, Mannheim, Germany). Complete blood counts were performed on a Sysmex XN-9000 automated haematology analyser (Sysmex Corporation, Kobe, Japan). HbA1c was measured by high-performance liquid chromatography (Bio-Rad Variant II Turbo, Bio-Rad Laboratories, Hercules, CA, USA). Bone-turnover and bone-metabolism markers (P1NP, C-terminal telopeptide of type I collagen [β-CTX], OC, BAP, 25-hydroxyvitamin D [25OHD], parathyroid hormone [PTH], calcitonin) were measured by electrochemiluminescence on the Roche cobas e601. No long-term storage was used; all assays were performed on freshly drawn samples on the day of collection. The full panel measured comprised: C-reactive protein (CRP), white blood cell count (WBC), HGB, haematocrit (HCT), mean corpuscular volume (MCV), platelet count (PLT), albumin (ALB), TG, high-density lipoprotein cholesterol (HDL), low-density lipoprotein cholesterol (LDL), total cholesterol (TC), creatinine, blood urea nitrogen (BUN), uric acid (UA), estimated glomerular filtration rate (eGFR), FPG, HbA1c, P1NP, β-CTX, OC, BAP, 25-OHD, PTH, calcitonin, Ca and serum phosphorus (P).

### 2.7 Exposure variables

Three indices were prespecified to capture distinct dimensions of the IR–adiposity–bone axis: TyG indexes the systemic IR signal alone; TyG-BMI incorporates total adiposity; and TyG-WC incorporates central (visceral) adiposity, hypothesized to be the most metabolically active and bone-deleterious adipose depot, as shown in Equations 1–3. This three-tier prespecification enables direct head-to-head comparison of incremental anthropometric contribution. The indices were computed as:

TyG=lnTG mg/dL×FPG mg/dL / 2 (1)

TyG−BMI=TyG×BMI kg/m2 (2)

TyG−WC=TyG×WC cm (3)

Where TG was originally measured in mmol/L, values were converted to mg/dL by multiplication by 88.57 before TyG calculation, to ensure consistency with the published TyG definition.

### 2.8 Statistical analysis

#### 2.8.1 Descriptive statistics

Continuous variables with approximately normal distribution were expressed as mean ± standard deviation (SD) and compared by Student’s t-test. Non-normally distributed variables, as median (interquartile range, IQR) and compared by Mann-Whitney U test. Categorical variables were expressed as n (%) and compared by chi-square test (or Fisher’s exact test where appropriate).

#### 2.8.2 Variable selection

Candidate variables with univariable P < 0.20 (Supplementary Table S1) entered a 10-fold cross-validated least absolute shrinkage and selection operator (LASSO) regression. Variables retained at λ-1SE (Supplementary Table S2) were carried forward; VIF (Supplementary Table S2) confirmed no concerning multicollinearity. Nine covariates were ultimately selected for inclusion based on previous literature and biological plausibility, LASSO and VIF analysis results: sex, age, HGB, HDL, HbA1c, P1NP, OC, BAP and Ca.

#### 2.8.3 Primary multivariable analysis

Because the events-per-variable ratio was low (36 events/nine covariates ≈4), Firth’s penalised-likelihood logistic regression was the primary analysis for each of three parallel models. The dependent variable was binary OP (yes/no). The primary independent variables of interest, entered separately, were TyG (Model 1), TyG-BMI (Model 2) and TyG-WC (Model 3). All three models were adjusted for the same nine covariates listed above.

#### 2.8.4 Sensitivity analyses

Two prespecified sensitivity analyses were performed: (a) conventional maximum-likelihood logistic regression (Supplementary Table S3); (b) ridge logistic regression with 10-fold cross-validation–selected λ (Supplementary Table S4). Estimates from both sensitivity analyses were directionally concordant with the Firth-penalised primary analysis.

#### 2.8.5 Discrimination, calibration and pairwise model comparison

Discrimination was quantified by area under the curve (AUC) with 95% CI computed by DeLong method. Pairwise AUC comparisons used DeLong’s test. Internal validation employed 1,000 bootstrap resamples to estimate optimism; apparent AUC, optimism, and optimism-corrected AUC are reported separately. Calibration was assessed by a calibration plot, intercept, slope, and Hosmer–Lemeshow goodness-of-fit test. Incremental discrimination over (i) the nine-covariate base model and (ii) each individual standard OP-related marker was quantified by the change in AUC and tested using DeLong (Lumley et al., 2002)

#### 2.8.6 Dose-response analyses

To examine the dose-response relationship between IR indices and OP risk, each index was additionally analysed as quartiles (Q1 referent) within Firth-penalised logistic regression with adjustment for the same nine covariates, with a test for linear trend across quartile medians. Non-linearity was further examined by restricted cubic splines with three knots placed at the 10th, 50th and 90th percentiles.

#### 2.8.7 Software and significance

Statistical analyses were performed using R 2025.01.1 + 513 (PBC). A two-tailed P < 0.05 was considered significant.

## 3 Results

### 3.1 Cohort characteristics and QCT-BMD distribution

**FIGURE 1:**
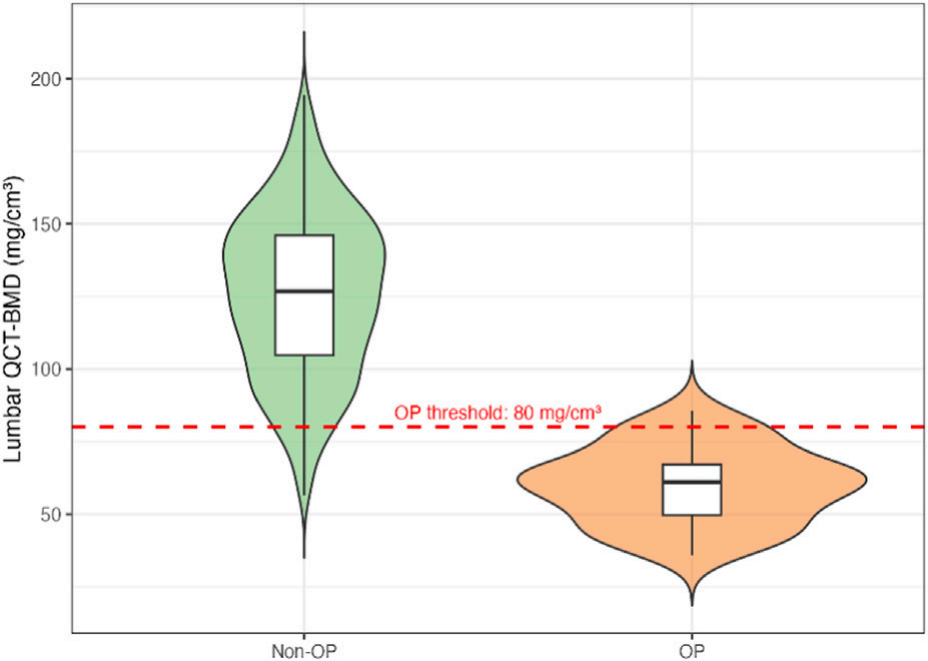
Distribution of BMD in the OP and non-OP groups.

A total of 568 patients with T2DM were included; 36 (6.3%) had QCT-defined OP and 532 (93.7%) did not (Table 1). OP patients differed from non-OP patients across anthropometric, glycaemic, lipid, haematological and bone-turnover dimensions, with the largest absolute separation in QCT (median 61.1 mg/cm^3^ [IQR 49.0–67.3] in OP vs. 126.8 mg/cm^3^ [IQR 104.6–146.0] in non-OP, P < 0.001) and HbA1c (7.7% vs. 6.7%, P < 0.001). The bimodal distribution of QCT with clear separation around the 80 mg/cm^3^ diagnostic threshold is illustrated in Figure 1. The OP group had a significantly higher proportion of women (61.1% vs. 35.3%, P = 0.002), consistent with the well-established sex-sensitivity of OP.

**TABLE 1:** Comparison of baseline characteristics of patients between the OP group and the non-OP group.

OP diagnosis	Total (n = 568)	Non-OP n = 532	OP n = 36	t/z/x^2^	p
QCT, mg/cm^3^	124.13 (98.48,144.46)	126.77 (104.60,145.98)	61.09 (48.95,67.31)	95.036	<0.001
Anthropometric and clinical measurements					
Sex, n (%)					
Female	210 (36.97)	188 (35.34)	22 (61.11)	9.611	0.002
Male	358 (63.03)	344 (64.66)	14 (38.89)		
Age, years	66 (57,73)	66 (57,73)	68 (65,75)	3.012	0.083
Systolic, mmHg	128 (116,144)	128 (116,144)	120 (115,143)	0.856	0.355
Diastolic, mmHg	76 (67,84)	76 (67,84)	77 (68,86)	0.626	0.429
Pulse, beats/min	72 (65,79)	72 (65,79)	72 (65,78)	0.279	0.598
WC, cm	82.50 (76.00,90.00)	81.85 (75.90,89.90)	89.00 (82.40,96.50)	15.444	<0.001
BMI, kg/m^2^	22.65 (20.42,25.12)	22.52 (20.32,24.92)	24.98 (22.93,27.11)	12.127	0.001
Biochemical and haematological markers					
CRP, mg/l	1.20 (0.60,2.30)	1.20 (0.60,2.35)	1.10 (0.65,2.15)	0.241	0.623
WBC, 10^9^/L	6.20 (5.10,7.40)	6.20 (5.10,7.50)	5.87 (4.78,7.10)	2.690	0.101
HGB, g/dl	14.50 (13.20,15.80)	14.50 (13.20,15.90)	13.75 (12.30,14.90)	8.069	0.005
HCT, %	41.80 (38.50,45.95)	41.80 (38.70,46.05)	41.30 (37.35,45.45)	1.477	0.224
MCV, fl	91.50 (86.65,95.40)	91.45 (86.70,95.45)	92.15 (85.70,94.65)	0.433	0.511
PLT, 10^9^/L	201.00 (159.50,233.50)	201.00 (161.00,233.00)	185.50 (132.50,238.50)	1.341	0.247
ALB, g/L	40.58 ± 4.57	40.60 ± 4.61	40.28 ± 4.08	0.413	0.680
TG, mg/dl	111.06 (81.42,161.42)	105.75 (79.65,156.64)	161.33 (116.82,222.30)	19.909	<0.001
HDL, mg/dl	53.28 (46.33,61.00)	54.05 (46.33,61.59)	49.62 (45.17,56.76)	5.190	0.023
LDL, mg/dl	104.83 (86.87,125.87)	104.83 (86.49,126.64)	103.09 (91.12,119.69)	0.056	0.813
TC, mg/dl	188.03 (166.61,213.90)	188.23 (166.61,214.48)	179.35 (166.02,201.93)	0.827	0.363
Cr, mg/dl	0.79 (0.66,0.91)	0.79 (0.66,0.91)	0.75 (0.64,0.89)	1.487	0.223
BUN, mg/dl	14.85 (12.04,17.93)	14.85 (12.04,18.21)	14.71 (11.34,16.53)	1.353	0.245
UA, mg/dL	5.00 (4.05,5.90)	5.00 (4.00,5.95)	4.95 (4.10,5.90)	0.008	0.930
eGFR, mL/min/1.73 m^2^	109.53 ± 32.91	109.40 ± 33.24	111.48 ± 27.84	−0.366	0.715
FPG, mg/dl	103.69 (96.49,112.43)	101.98 (95.59,110.90)	115.50 (105.59,120.90)	21.620	<0.001
HbA1c, %	6.80 (6.50,7.10)	6.70 (6.50,7.05)	7.70 (7.25,8.15)	59.877	<0.001
P1NP, ng/mL	41.89 (30.22,53.47)	41.09 (30.07,52.70)	46.12 (39.19,57.34)	5.332	0.021
β-CTX, ng/mL	0.27 (0.15,0.38)	0.27 (0.14,0.38)	0.27 (0.17,0.37)	0.057	0.812
OC, ng/mL	11.70 ± 4.71	11.56 ± 4.63	13.87 ± 5.35	−2.867	0.004
BAP, ug/L	16.02 ± 6.01	15.81 ± 5.83	19.19 ± 7.63	−3.290	0.001
25OHD, ng/mL	18.97 ± 7.75	19.01 ± 7.79	18.35 ± 7.25	0.495	0.621
PTH, pg/ml	42.73 ± 17.16	42.96 ± 16.88	42.28 ± 20.14	0.234	0.815
Calcitonin, pg/ml	1.89 (0.91,2.96)	1.89 (0.91,2.98)	1.86 (0.90,2.84)	0.225	0.635
Ca, mmol/L	2.28 ± 0.14	2.29 ± 0.14	2.24 ± 0.17	1.875	0.061
P, mmol/L	1.11 ± 0.19	1.11 ± 0.19	1.12 ± 0.21	−0.234	0.815
IR related indicators					
TyG	8.64 (8.31,9.09)	8.61 (8.29,9.04)	9.10 (8.81,9.56)	20.143	<0.001
TyG-BMI	195.41 (174.41,223.86)	193.95 (172.99,221.08)	226.79 (201.61,251.63)	20.424	<0.001
TyG-WC	716.86 (648.09,803.58)	710.80 (644.33,795.08)	817.13 (734.29,918.80)	22.577	<0.001

Continuous variables with normal distribution were expressed as mean ± standard deviation, and t-test was used. Non-normal distribution was expressed as median (interquartile range), and Mann-Whitney U test was used. Categorical variables were expressed as frequencies (percentages) with the use of the chi-square test. WC, waist circumference; BMI, body mass index; QCT, quantitative computed tomography; CRP C-reactive protein; WBC, white blood cell count; HGB, hemoglobin; HCT, hematocrit; MCV, mean corpuscular volume; PLT, platelet count; ALB, Blood biochemical: albumin; TG, triglyceride; HDL, high density lipoprotein cholesterol; LDL, low density lipoprotein cholesterol; TC, total cholesterol, Cr creatinine; BUN, blood urea nitrogen; UA, uric acid; eGFR, estimated Glomerular Filtration Rate; FPG, fasting plasma glucose; HbA1c glycosylated hemoglobin; P1NP N-terminal propeptide of type I procollagen; β-CTX C-terminal telopeptide of type I collagen; OC, osteocalcin; BAP, bone-specific alkaline phosphatase; 25OHD, 25-hydroxyvitamin D; PTH, parathyroid hormone, Ca serum calcium, P serum phosphorus; TyG triglyceride–glucose.

### 3.2 Insulin-resistance indices in the two groups

All three IR-related indices were substantially higher in OP than non-OP patients: TyG median 9.10 (IQR 8.81–9.56) vs. 8.61 (8.29–9.04); TyG-BMI 226.79 (201.61–251.63) vs. 193.95 (172.99–221.08); TyG-WC 817.13 (734.29–918.80) vs. 710.80 (644.33–795.08); all P < 0.001 (Table 1).

**FIGURE 2:**
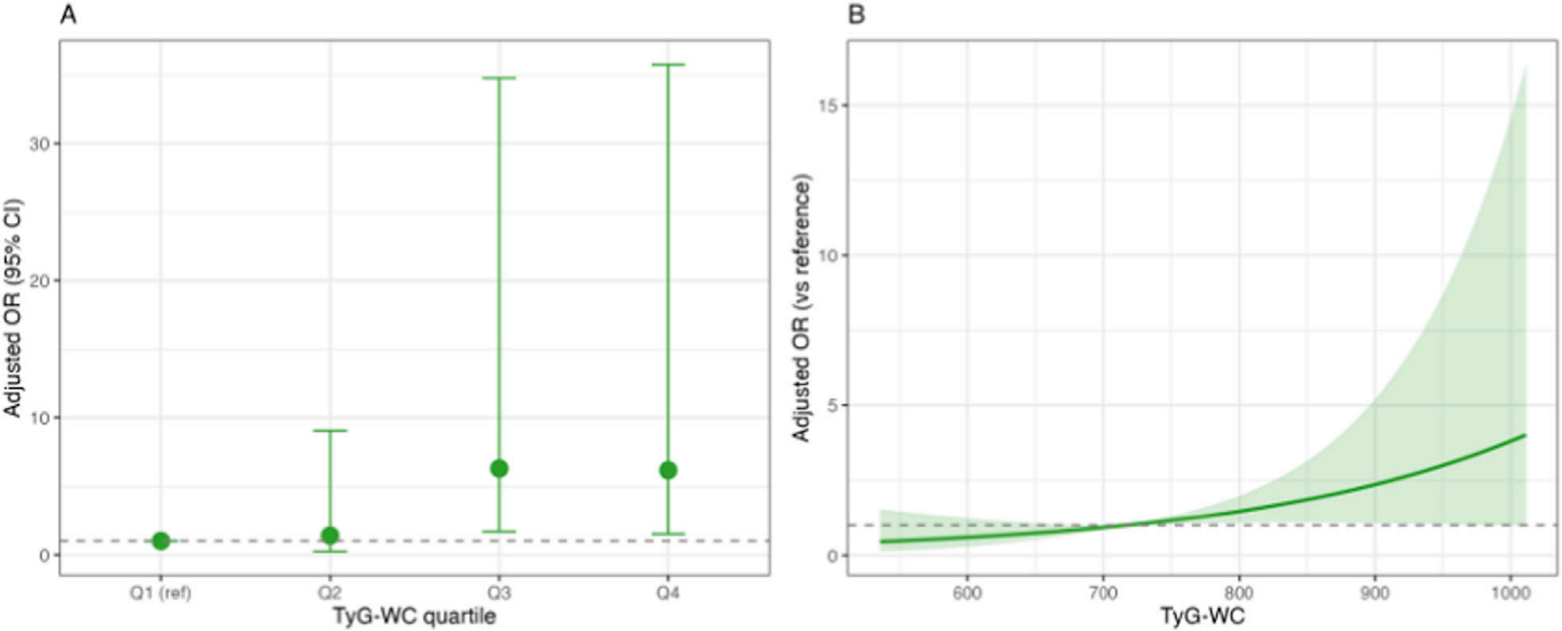
Dose-response relationship between TyG-WC and adjusted odds of OP. **(A)** Quartile-based dose-response, **(B)** Restricted cubic spline.

A clear dose-response relationship was observed for TyG-WC. When TyG-WC was analysed in quartiles, the Firth-penalised adjusted OR for OP increased monotonically from Q1 (referent) through Q4 (trend P = 0.003), and restricted-cubic-spline analysis confirmed an approximately linear positive relationship (Figure 2). Parallel quartile and spline analyses for TyG and TyG-BMI are reported in Supplementary Figure S1.

### 3.3 Univariate and multivariate analyses of OP risk

In Supplementary Table S1, univariate analysis showed that TyG and TyG-WC were both significantly associated with OP (both P < 0.01), TyG-BMI showed a borderline association (P = 0.097). In addition, female, WC, HGB, TG, HDL, FPG, HbA1c, P1NP, OC, BAP, Ca, and QCT were also associated with OP risk (P < 0.05). Supplementary Table S2 shown LASSO regression to identify a parsimonious covariate set. Based on previous literature combined with univariate and LASSO results, sex, age, HGB, HDL, HbA1c, P1NP, OC, BAP, and Ca were retained in. All retained covariates had low VIF values, indicating no significant multicollinearity, as shown in Supplementary Table S2.

### 3.4 Multivariable firth-penalised analyses

In the Firth-penalised multivariable models (Table 2), only TyG-WC remained independently associated with OP (Model 3: OR 1.004 per unit, 95% CI 1.001–1.008, P = 0.013). Neither TyG nor TyG-BMI reached statistical significance after adjustment, although both retained positive, directionally consistent point estimates. Across all three models, female and higher HGB were independently protective, whereas higher HbA1c, OC and BAP independently increased the odds of OP. Maximum-likelihood and ridge sensitivity analyses (Supplementary Tables S3–S4) gave directionally concordant and quantitatively similar estimates.

**TABLE 2:** Multivariable Logistic regression models for predicting the risk of OP in patients with T2DM.

	Model 1:TyG		Model 2:TyG-BMI		Model 3:TyG-WC	
Variable	OR (95%CI)	P	OR (95%CI)	P	OR (95%CI)	P
TyG	1.871 (0.938–3.819)	0.075				
TyG-BMI			1.002 (0.999–1.005)	0.105		
TyG-WC					1.004 (1.001–1.008)	0.013
Age	0.999 (0.967–1.034)	0.946	1.004 (0.973–1.038)	0.797	0.994 (0.962–1.029)	0.735
Sex						
Female	Ref.		Ref.		Ref.	
Male	0.305 (0.134–0.661)	0.003	0.306 (0.135–0.661)	0.002	0.293 (0.127–0.641)	0.002
HGB	0.710 (0.571–0.861)	0.000	0.707 (0.574–0.857)	0.000	0.681 (0.547–0.831)	0.000
HDL	0.979 (0.942–1.017)	0.281	0.971 (0.936–1.005)	0.092	0.979 (0.942–1.015)	0.251
HbA1c	1.848 (1.326–2.633)	0.000	2.151 (1.618–2.926)	0.000	1.845 (1.339–2.586)	0.000
P1NP	1.018 (0.994–1.042)	0.143	1.019 (0.995–1.044)	0.117	1.017 (0.994–1.042)	0.148
OC	1.117 (1.031–1.218)	0.007	1.105 (1.021–1.201)	0.013	1.121 (1.033–1.222)	0.006
BAP	1.118 (1.052–1.195)	0.000	1.116 (1.050–1.192)	0.000	1.129 (1.060–1.211)	0.000
Ca	0.084 (0.005–1.162)	0.065	0.080 (0.005–1.147)	0.063	0.091 (0.006–1.291)	0.077

OR: odds ratio; CI: Confidence interval. HGB, hemoglobin; HDL, high density lipoprotein cholesterol; HbA1c glycosylated hemoglobin; P1NP N-terminal propeptide of type I procollagen; OC, osteocalcin; BAP, bone-specific alkaline phosphatase; Ca serum calcium, TyG triglyceride–glucose.

### 3.5 Receiver-operating-characteristic (ROC) analysis and pairwise comparison

**FIGURE 3:**
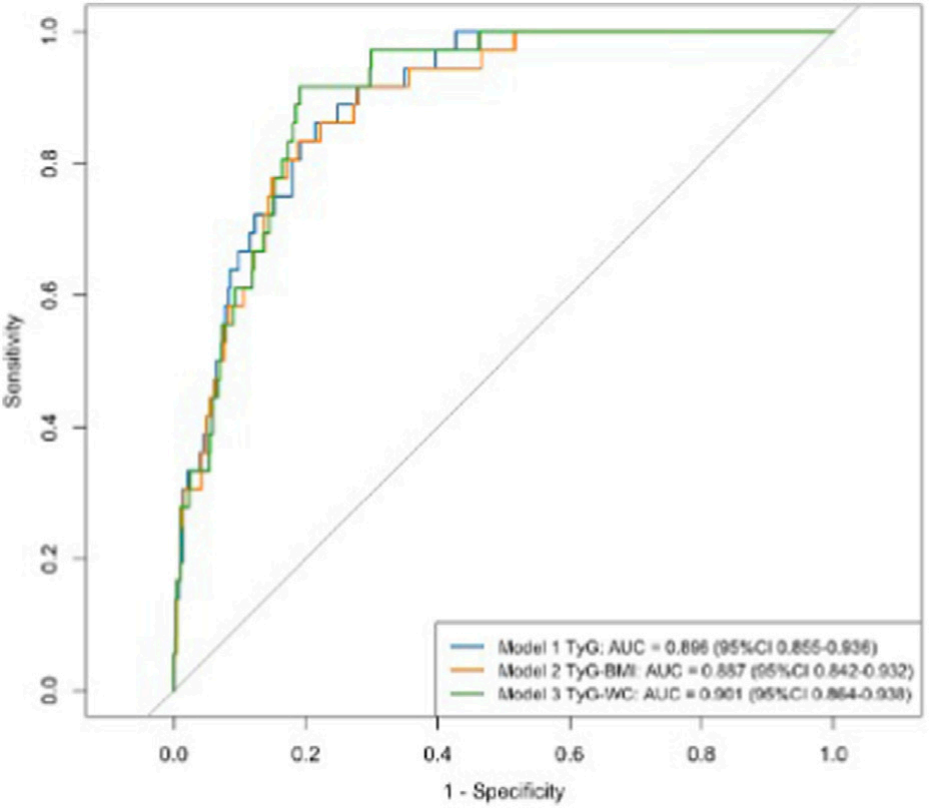
ROC curves of the three Firth-penalised multivariable models predicting OP in T2DM patients.

Apparent AUCs were 0.896 (0.856–0.936) forTyG, 0.887 (0.842–0.932) for TyG-BMI, and 0.901 (0.864–0.938) for TyG-WC; ROC curves are shown in Figure 3. The Youden-index optimal cut-points were 0.071 for TyG-WC, with sensitivity 91.67% and specificity 81.02%; corresponding cut-points for TyG-BMI and TyG are reported in Supplementary Table S5.

Pairwise DeLong tests did not establish statistically significant superiority of any one index: TyG-WC vs. TyG-BMI (ΔAUC 0.014, P = 0.141); TyG-WC vs. TyG (ΔAUC 0.005, P = 0.509); TyG-BMI vs. TyG (ΔAUC −0.008, P = 0.205) (Table 3). Optimism-corrected AUCs from 1,000 bootstrap resamples were 0.866, 0.860, and 0.870 for Models 1, 2 and 3, respectively; calibration was acceptable for all three models (calibration slope = 1.06–1.07, intercept <0.001; Hosmer–Lemeshow P = 0.0.843–0.900; full calibration plots in Supplementary Figure S2).

**TABLE 3:** Discrimination, internal validation and pairwise DeLong comparison.

Model	Apparent AUC (95% CI)	Optimism-corrected AUC	Calibration			
			Slope	Intercept	H_L	P
Model 1: TyG	0.896 (0.856–0.936)	0.866	1.060	<0.001	4.154	0.843
Model 2: TyG-BMI	0.887 (0.842–0.932)	0.860	1.070	<0.001	4.059	0.852
Model 3: TyG-WC	0.901 (0.864–0.938)	0.870	1.070	<0.001	3.495	0.900

Pairwise DeLong tests for ΔAUC		
Comparison	ΔAUC	DeLong P
TyG-WC vs. TyG	0.005	0.509
TyG-WC vs. TyG-BMI	0.014	0.141
TyG-BMI vs. TyG	−0.008	0.205

### 3.6 Incremental discrimination over base model and standard markers

To address whether the IR indices add value beyond established OP-related markers, we compared the three IR-index models against (i) a baseline model containing only the nine adjustment covariates, and (ii) each individual standard marker used alone. TyG-WC retained the numerically highest discrimination across all comparisons, although the incremental gains were modest and did not reach DeLong significance versus HbA1c alone (Table 4).

**TABLE 4:** Incremental discrimination of full TyG-WC model over individual standard OP-related markers.

Single-marker model	AUC (95% CI)	ΔAUC vs. TyG-WC model	DeLong P (vs. TyG-WC)
HbA1c alone	0.885 (0.845–0.925)	0.016	0.514
FPG alone	0.731 (0.660–0.802)	0.170	<0.001
BMI alone	0.673 (0.585–0.761)	0.228	<0.001
WC alone	0.696 (0.607–0.785)	0.206	<0.001
OC alone	0.628 (0.529–0.728)	0.273	<0.001
BAP alone	0.630 (0.525–0.734)	0.272	<0.001
HGB alone	0.641 (0.556–0.726)	0.260	<0.001
TyG alone (univariable)	0.734 (0.670–0.799)	0.167	<0.001
TyG-BMI alone (univariable)	0.723 (0.643–0.804)	0.178	<0.001
TyG-WC alone (univariable)	0.736 (0.654–0.819)	0.165	<0.001
Full TyG-WC model (model 3)	0.901 (0.864–0.938)	Ref	—

Univariable AUCs are for each marker entered alone. The full TyG-WC model (Model 3) is the Firth-penalised multivariable model with TyG-WC and the nine adjustment covariates. DeLong P-values test the null hypothesis of no difference in AUC versus Model 3.

## 4 Discussion

### 4.1 Principal findings

In 568 adults with T2DM (36 OP events), all three IR surrogates were significantly higher in OP patients at the univariable level. After Firth-penalised multivariable adjustment, however, only TyG-WC remained independently associated with OP, while TyG and TyG-BMI showed positive but non-significant association. A clear quartile and spline-based dose-response relationship was observed for TyG-WC. Discrimination was high, but DeLong pairwise tests did not establish significant superiority of any one index. Incremental gain of TyG-WC over a base model containing HbA1c and bone-turnover markers was modest.

### 4.2 IR, visceral adiposity and bone: mechanistic interpretation

The observation that only the central-adiposity-integrated index (TyG-WC), rather than systemic IR alone (TyG) or total adiposity (TyG-BMI), reached independent significance is biologically coherent. WC is the anthropometric proxy for visceral adipose tissue (VAT), the depot most directly implicated in bone-deleterious endocrine signalling (Pouliot et al., 1994). VAT secretes elevated levels of TNF-α and IL-6, produces less adiponectin and more leptin; these adipokine imbalances promote osteoclastogenesis through RANKL upregulation and suppress osteoblast differentiation through WNT/β-catenin antagonism (Oliveira et al., 2020). At the cellular level, IR independently impairs osteoblast IGF-1 responsiveness and induces osteocyte senescence (Ravindran et al., 2025). TyG-WC multiplies a systemic IR (TyG) and visceral adiposity (WC), and this combination may capture a composite signal that neither TyG nor BMI-scaled TyG could resolve in our cohort. This interpretation should nonetheless be made cautiously, because the between-model AUC difference were small and not statistically significant on DeLong testing.

### 4.3 HbA1c and bone-turnover markers in diabetic OP

HbA1c was a strong independent predictor of OP across all three models, consistent with the well-established toxicity of chronic hyperglycaemia on bone via advanced glycation end-product (AGE) accumulation in collagen, which increases brittleness independently of BMD (Chiodini et al., 2021). The independent positive association of bone-formation markers (OC, BAP) with OP, though counter-intuitive at first glance, is consistent with the increasingly recognised ‘low-but-uncoupled’ bone-turnover phenotype of diabetic OP, in which compensatory or disordered turnover signals coexist with poor bone quality (Chen and Dai, 2021; Højsager et al., 2019; Liu et al., 2017; Fusaro et al., 2020). Bone-turnover markers in T2DM should therefore be interpreted in conjunction with imaging and not as stand-alone risk indicators.

### 4.4 Comparison with prior literature

Our findings extend prior work on TyG-related indices and bone outcomes (Pan et al., 2023; Li et al., 2024; Xu J. et al., 2024; Kahn et al., 2006; Gkastaris et al., 2020; King et al., 2022; Tian et al., 2024; Wen et al., 2022). Compared with published general-population NHANES analyses (typical AUCs 0.65–0.75) (Tian et al., 2024; Lai et al., 2025), the absolute AUCs in our T2DM-enriched cohort are higher (0.89–0.90). This difference is largely attributable to the discriminative power of the adjustment set (HbA1c and bone-turnover markers) rather than to a stronger IR signal alone; the incremental AUC contributed specifically by the IR index over the base model is more modest and is the more honest performance metric. Compared with the Kailuan prospective cohort (Yao et al., 2025), which reported a linear dose-response between cumulative TyG and incident fragility fractures, our cross-sectional dose-response findings for TyG-WC are consistent in direction. The Pan et al. study (Pan et al., 2023) in postmenopausal women with T2DM and OP reported similar TyG–fracture associations. The novelty of the present work lies in (i) the explicit head-to-head DeLong-based comparison of three TyG variants, (ii) the use of Firth’s penalised regression to address the low EPV that has plagued prior analyses, and (iii) the explicit reporting of incremental AUC over both a multivariable base model and standard single markers. Notably, in our adjusted analysis only TyG-WC reached independent significance, suggesting that the visceral-adiposity component, rather than systemic IR or total adiposity, carries the residual skeletal signal in this population.

### 4.5 Clinical implications

TyG-WC derived from routinely available fasting lipids and glucose together with a simple tape measurement, may serve as low-cost adjuncts for identifying T2DM patients who warrant earlier QCT assessment. Because the discrimination of TyG-WC did not significantly exceed that of TyG, TyG-BMI, or HbA1c alone, and its incremental AUC over a model already containing HbA1c and bone-turnover markers was modest, we do not recommend its adoption as a sole screening tool on the basis of these single-centre data. Where an anthropometry-augmented IR index is used pragmatically, TyG-WC is the variant with the strongest adjusted association in our cohort, but the choice may also be guided by which anthropometric measurement is more reliably available at the local clinic.

### 4.6 Strengths and limitations

Strengths include the use of QCT-defined OP, which is less susceptible than DXA to artefactual elevation in T2DM; a complete bone-turnover-marker panel; prespecified Firth’s penalised-likelihood regression to address low EPV; formal DeLong-based AUC comparisons; dose-response analyses by quartiles and restricted cubic splines; and bootstrap internal validation with optimism correction.

Several limitations require explicit acknowledgement. First, the cross-sectional design precludes causal inference. Second, only 36 events were available; even after Firth penalisation, this yields wide confidence intervals and the absolute AUC differences between models (≤0.014) were not DeLong-significant, so the apparent ranking among the three IR indices should be regarded as exploratory. Third, this is a single-centre tertiary cohort, limiting generalizability to community settings; multi-centre prospective validation is required. Fourth, no non-diabetic control group was included, by design: the present study addresses risk stratification within T2DM rather than the broader question of whether IR–OP associations are specific to T2DM versus operating generically across diabetic and non-diabetic populations. Future studies recruiting paired diabetic and non-diabetic cohorts are warranted to address that distinct question. Fifth, residual confounding remains possible because information on several relevant covariates (e.g., diabetes duration, antidiabetic and anti-osteoporotic medications, menopausal status, vitamin D supplementation, physical activity and fall history) was incomplete or unavailable in this retrospective dataset; the expected direction of the resulting bias is mixed, and the adjusted point estimates should be interpreted as upper bounds. Sixth, although LASSO mitigates model-selection uncertainty, residual two-step selection bias cannot be excluded. Finally, the composite indices (TyG-BMI, TyG-WC) integrate two signals whose individual contributions are not uniquely identifiable.

### 4.7 Conclusions

In a single-centre cohort of adults with T2DM, TyG-WC—the insulin-resistance surrogate that integrates central adiposity—was the only index independently associated with QCT-defined OP after small-sample-bias correction; TyG and TyG-BMI showed positive but non-significant trends, and overall discrimination was similar across all three indices. These hypothesis-generating findings require prospective external validation in larger, multi-centre and ideally paired diabetic/non-diabetic cohorts, with richer covariate data, before any clinical recommendation about TyG-related indices for OP screening in T2DM can be made.

## Data Availability

The raw data supporting the conclusions of this paper are provided by Shanghai Pudong Hospital (Pudong Hospital Affiliated to Fudan University), further inquiries need to apply to hospital for approval. Research data are not shared. Requests to access the datasets should be directed to Q H, jackkai2012@163.com.
