# Supplementary material for: Predictive value of insulin resistance related indicators for osteoporosis risk in patients with type 2 diabetes mellitus: a real-world study
Source: Front Mol Biosci. 2026 Jul 22;13:1795616. doi: 10.3389/fmolb.2026.1795616 (PMC13437509; doi:10.3389/fmolb.2026.1795616)
Supplement: Supplementary file 1 [file Table1.docx]

**Supplementary Materials**

**Supplementary Methods. Post hoc statistical-power estimate**

Because this was a retrospective all-comer study without an a priori sample-size calculation (Main text, Section 2.3), we performed a post hoc power estimate for the achieved sample using the method of Hsieh et al. (1998) for logistic regression with a continuous predictor. For a single continuous exposure, the required sample size (and, by rearrangement, the achievable power) is given by:

*n = (Z₁₋α/₂ + Z₁₋β)² / [ p (1 − p) (β*)² ] × 1/(1 − ρ²)	(Eq. S1)*

where p is the marginal event probability (here 36/568 = 0.063), β* = ln(OR per 1-SD increase in the exposure), Z₁₋α/₂ = 1.96 for a two-sided α of 0.05, and ρ² is the proportion of variance in the exposure explained by the remaining covariates (the variance-inflation correction for multivariable models). Rearranging Eq. S1 to solve for Z₁₋β yields the achieved power, Φ(Z₁₋β).

For TyG-WC, the standard deviation in the full cohort was approximately 115 units; the univariable odds ratio of 1.006 per unit corresponds to an OR of approximately 2.0 per 1-SD increase (β* ≈ 0.69). Substituting these values (and conservatively allowing ρ² = 0.20 for the multivariable correction) gives an achieved power exceeding 95% at α = 0.05. The achievable power was correspondingly lower for indices with weaker standardized effect sizes (TyG and TyG-BMI), which is consistent with their non-significant associations in the adjusted models. These estimates indicate that the study was adequately powered to detect the TyG-WC–OP association observed, while reinforcing that the non-significant findings for TyG and TyG-BMI may partly reflect limited power at the available event count. All power calculations should be interpreted as approximate, given the post hoc nature and the modest number of events.

**Post hoc power estimates for the three IR indices (Hsieh 1998 method)**

| **Index** | **OR per unit*** | **Approx. OR per 1 SD** | **β* = ln(OR/SD)** | **Approx. power (α = 0.05)** |
| --- | --- | --- | --- | --- |
| TyG | 2.750 | ≈2.0† | ≈0.69 | >90% |
| TyG-BMI | 1.002 | ≈1.5 | ≈0.41 | ≈70–80% |
| TyG-WC | 1.006 | ≈2.0 | ≈0.69 | >95% |

*Univariable OR (Supplementary Table S1). †TyG is reported per natural-log unit; its standard deviation is small, so the OR per 1-SD increase is reported on the standardized scale for comparability. Power values are approximate post hoc estimates from Eq. S1 with a variance-inflation correction of ρ² = 0.20; they are provided for transparency and should not be interpreted as a priori sample-size justification. The primary mitigation of the low events-per-variable ratio is the use of Firth's penalised-likelihood regression (Main text, Section 2.8.3).

**Supplementary Table S1 Full univariable logistic regression of every candidate variable for OP.**

|  | OR | SE | z | P>z | 95%CI | |
| --- | --- | --- | --- | --- | --- | --- |
| QCT | 0.856 | 0.021 | -6.410 | 0.000 | 0.816 | 0.898 |
| Male | 0.348 | 0.354 | -2.990 | 0.003 | 0.170 | 0.689 |
| Age | 1.018 | 0.013 | 1.450 | 0.148 | 0.994 | 1.044 |
| Systolic, mmHg | 0.994 | 0.008 | -0.700 | 0.485 | 0.978 | 1.011 |
| Diastolic, mmHg | 1.008 | 0.014 | 0.590 | 0.554 | 0.981 | 1.036 |
| Pulse, beats/min | 1.015 | 0.016 | 0.910 | 0.361 | 0.983 | 1.047 |
| WC, cm | 1.062 | 0.018 | 3.540 | 0.000 | 1.027 | 1.097 |
| BMI (kg/m2) | 1.013 | 0.012 | 1.110 | 0.265 | 0.990 | 1.036 |
| CRP, mg/l | 0.954 | 0.070 | -0.640 | 0.522 | 0.826 | 1.102 |
| WBC, 10^9^/L | 0.846 | 0.088 | -1.610 | 0.108 | 0.690 | 1.037 |
| HGB, g/dl | 0.779 | 0.069 | -2.820 | 0.005 | 0.655 | 0.926 |
| HCT, % | 0.955 | 0.030 | -1.470 | 0.143 | 0.899 | 1.016 |
| MCV, fl | 0.986 | 0.016 | -0.830 | 0.408 | 0.955 | 1.019 |
| PLT, 10^9^/L | 0.995 | 0.003 | -1.710 | 0.087 | 0.990 | 1.001 |
| ALB (g/L) | 0.984 | 0.037 | -0.410 | 0.679 | 0.914 | 1.060 |
| TG, mg/dl | 1.004 | 0.001 | 3.240 | 0.001 | 1.002 | 1.007 |
| HDL, mg/dl | 0.967 | 0.015 | -2.150 | 0.032 | 0.938 | 0.997 |
| LDL, mg/dl | 0.998 | 0.006 | -0.360 | 0.716 | 0.986 | 1.009 |
| TC, mg/dl | 0.995 | 0.005 | -0.950 | 0.341 | 0.986 | 1.005 |
| Cr, mg/dl | 0.753 | 0.587 | -0.360 | 0.716 | 0.164 | 3.470 |
| BUN, mg/dl | 0.941 | 0.039 | -1.440 | 0.150 | 0.867 | 1.022 |
| UA, mg/dL | 0.941 | 0.114 | -0.500 | 0.614 | 0.743 | 1.192 |
| eGFR, mL/min/1.73 m2 | 1.002 | 0.005 | 0.370 | 0.714 | 0.992 | 1.012 |
| FPG, mg/dl | 1.009 | 0.004 | 2.490 | 0.013 | 1.002 | 1.017 |
| HbA1c, % | 1.931 | 0.247 | 5.140 | 0.000 | 1.503 | 2.481 |
| P1NP, ng/mL | 1.023 | 0.010 | 2.220 | 0.026 | 1.003 | 1.044 |
| β-CTX, ng/mL | 1.488 | 1.579 | 0.370 | 0.708 | 0.186 | 11.914 |
| OC, ng/mL | 1.112 | 0.042 | 2.820 | 0.005 | 1.033 | 1.197 |
| BAP, ug/L | 1.098 | 0.032 | 3.210 | 0.001 | 1.037 | 1.162 |
| 25OHD, ng/mL | 0.989 | 0.022 | -0.500 | 0.620 | 0.947 | 1.033 |
| PTH, pg/ml | 0.998 | 0.010 | -0.230 | 0.815 | 0.978 | 1.018 |
| Calcitionin, pg/ml | 0.914 | 0.111 | -0.740 | 0.461 | 0.720 | 1.161 |
| Ca, mmol/L | 0.106 | 0.128 | -1.860 | 0.063 | 0.010 | 1.125 |
| P, mmol/L | 1.242 | 1.146 | 0.230 | 0.815 | 0.203 | 7.580 |
| TyG | 2.750 | 0.646 | 4.310 | 0.000 | 1.736 | 4.357 |
| TyG-BMI | 1.002 | 0.001 | 1.660 | 0.097 | 1.000 | 1.005 |
| TyG-WC | 1.006 | 0.001 | 4.520 | 0.000 | 1.003 | 1.009 |

Note: WC waist circumference; BMI Body mass index; QCT quantitative computed tomography; CRP C-reactive protein; WBC white blood cell count; HGB hemoglobin; HCT hematocrit, MCV mean corpuscular volume; PLT platelet count; ALB Blood biochemical: albumin; TG triglyceride; HDL high density lipoprotein cholesterol; LDL low density lipoprotein cholesterol; TC total cholesterol, Cr creatinine; BUN blood urea nitrogen; UA uric acid; eGFR estimated Glomerular Filtration Rate; FPG fasting plasma glucose; HbA1c glycosylated hemoglobin;P1NP N-terminal propeptide of type I procollagen; β-CTX C-terminal telopeptide of type I collagen; OC osteocalcin; BAP bone-derived alkaline phosphatase; 25OHD 25-hydroxyvitamin D; PTH parathyroid hormone, Ca serum calcium, P serum phosphorus; TyG triglyceride–glucose.

**Supplementary Table S2 Lasso analysis and Variance inflation factors (VIF) for the retained variables**

|  | |  | **Lasso analysis results** | **VIF test results** |
| --- | --- | --- | --- | --- |
| **Variables** | **P>0.20 in univariate analysis** | | **λ-1se** | **VIF** |
| **Age** | 0.148 | |  |  |
| **Sex** | 0.003 | | 0.285 | 1.395 |
| **WC** | 0.000 | | 0.003 | 1.580 |
| **WBC** | 0.108 | | -0.008 |  |
| **HGB** | 0.005 | | -0.059 |  |
| **HCT** | 0.143 | | . |  |
| **PLT** | 0.087 | | -0.002 |  |
| **BUN** | 0.150 | | . |  |
| **TG** | 0.001 | | 0.000 | 1.040 |
| **HDL** | 0.032 | | . |  |
| **FPG** | 0.013 | | . |  |
| **HbA1c** | 0.000 | | 0.487 | 2.303 |
| **P1NP** | 0.026 | | . |  |
| **OC** | 0.005 | | 0.011 | 1.672 |
| **BAP** | 0.001 | | 0.032 | 2.107 |
| **Ca** | 0.063 | |  |  |

**Supplementary Table S3 Sensitivity analysis: conventional maximum-likelihood logistic regression.**

|  | Model 1：TyG | | Model 2：TyG-BMI | | Model 3：TyG-WC | |
| --- | --- | --- | --- | --- | --- | --- |
|  | OR (95%CI) | P | OR (95%CI) | P | OR (95%CI) | P |
| TyG | 1.956(0.957-4.096) | 0.069 |  |  |  |  |
| TyG-BMI |  |  | 1.002(0.997-1.005) | 0.210 |  |  |
| TyG-WC |  |  |  |  | 1.005(1.001-1.008) | 0.013 |
| Age | 1.000(0.967-1.036) | 0.998 | 1.006(0.974-1.041) | 0.728 | 0.995(0.962-1.031) | 0.769 |
| Sex |  |  |  |  |  |  |
| Female | Ref. |  | Ref. |  | Ref. |  |
| Male | 0.281(0.119-0.626) | 0.002 | 0.284(0.121-0.629) | 0.002 | 0.270(0.113-0.608) | 0.002 |
| HGB | 0.692(0.552-0.847) | 0.001 | 0.693(0.558-0.847) | 0.000 | 0.664(0.528-0.818) | 0.000 |
| HDL | 0.978(0.940-1.017) | 0.272 | 0.968(0.932-1.003) | 0.083 | 0.977(0.939-1.015) | 0.236 |
| HbA1c | 1.923(1.359-2.781) | 0.000 | 2.259(1.674-3.121) | 0.000 | 1.913(1.370-2.719) | 0.000 |
| P1NP | 1.019(0.995-1.044) | 0.137 | 1.020(0.996-1.046) | 0.112 | 1.018(0.995-1.044) | 0.139 |
| OC | 1.126(1.035-1.231) | 0.007 | 1.111(1.024-1.211) | 0.014 | 1.130(1.038-1.236) | 0.006 |
| BAP | 1.128(1.059-1.209) | 0.000 | 1.124(1.056-1.204) | 0.000 | 1.140(1.067-1.226) | 0.000 |
| Ca | 0.068(0.004-1.036) | 0.059 | 0.066(0.004-1.031) | 0.058 | 0.075(0.004-1.168) | 0.070 |

**Supplementary Table S4 Sensitivity analysis: ridge logistic regression with cross-validation-selected λ.**

|  | Model 1：TyG | Model 2：TyG-BMI | Model 3：TyG-WC |
| --- | --- | --- | --- |
|  | OR | OR | OR |
| TyG | 1.643 |  |  |
| TyG-BMI |  | 1.002 |  |
| TyG-WC |  |  | 1.003 |
| Age | 1.004 | 1.006 | 1.002 |
| Sex |  |  |  |
| Female | Ref. | Ref. | Ref. |
| Male | 0.613 | 0.679 | 0.606 |
| HGB | 0.793 | 0.858 | 0.769 |
| HDL | 0.986 | 0.986 | 0.984 |
| HbA1c | 1.686 | 1.659 | 1.71 |
| P1NP | 1.013 | 1.01 | 1.013 |
| OC | 1.081 | 1.053 | 1.086 |
| BAP | 1.083 | 1.056 | 1.09 |
| Ca | 0.18 | 0.297 | 0.175 |

**Supplementary Table S5 Youden-index optimal cut-points and corresponding sensitivities and specificities for the three IR indices.**

|  | **threshold** | **sensitivity** | **specificity** |
| --- | --- | --- | --- |
| **TyG** | 0.063 | 0.861 | 0.786 |
| **TyG-BMI** | 0.070 | 0.833 | 0.812 |
| **TyG-WC** | 0.071 | 0.917 | 0.810 |

**Supplementary Figure S1. Quartile and restricted-cubic-spline dose-response plots for TyG and TyG-BMI.**

**Supplementary Figure S2. Calibration plots for Models 1–3.**
